# A Mathematical Model of Interstitial Fluid Flow and Retinal Tissue Deformation in Macular Edema

**DOI:** 10.1167/iovs.65.11.19

**Published:** 2024-09-10

**Authors:** Alessia Ruffini, Mariia Dvoriashyna, Andrea Govetto, Mario R. Romano, Rodolfo Repetto

**Affiliations:** 1 Department of Civil, Chemical and Environmental Engineering, University of Genoa, Genoa, Italy; 2 School of Mathematics, University of Edinburgh, Edinburgh, United Kingdom; 3 Department of Biomedical Sciences, Humanitas University, Milan, Italy

**Keywords:** fluid flow, retinal tissue deformation, mathematical modeling, Müller cells, exudative macular edema

## Abstract

**Purpose:**

This study aims to develop a mathematical model to elucidate fluid circulation in the retina, focusing on the movement of interstitial fluid (comprising water and albumin) to understand the mechanisms underlying exudative macular edema (EME).

**Methods:**

The model integrates physiological factors such as retinal pigment epithelium (RPE) pumping, osmotic pressure gradients, and tissue deformation. It accounts for spatial variability in hydraulic conductivity (HC) across the retina and incorporates the structural role of Müller cells (MCs) in maintaining retinal stability.

**Results:**

The model predicts that tissue deformation is maximal at the center of the fovea despite fluid exudation from blood capillaries occurring elsewhere, aligning with clinical observations. Additionally, the model suggests that spatial variability in HC across the thickness of the retina plays a protective role against fluid accumulation in the fovea.

**Conclusions:**

Despite inherent simplifications and uncertainties in parameter values, this study represents a step toward understanding the pathophysiology of EME. The findings provide insights into the mechanisms underlying fluid dynamics in the retina and fluid accumulation in the foveal region, showing that the specific conformation of Müller cells is likely to play a key role.

The retina, a thin and highly specialized multilayered neural tissue located at the back of the eye, comprises a diverse array of cell types and structural elements, such as ganglion cells, blood vessels, amacrine cells, Müller cells (MCs), photoreceptors, and more. Structural stability of the retina and resistance to mechanical stress are guaranteed by MCs, which span nearly the whole thickness of the retina^1^ from the internal limiting membrane (ILM), which is their base membrane, to the external limiting membrane (ELM).^2^ The healthy retina is maintained at a highly controlled level of dehydration, ensuring maximum light transmission, thanks to the control of various active and passive mechanisms.^3^ In particular, active pumping of water and ions from the vitreous chamber to the choroid by the RPE plays a key role.^4^^,^^5^ Thus, in physiological conditions, fluid entry and exit into the retinal tissue are tightly regulated to maintain a balanced hydration state, compatible with retinal homeostasis and necessary for tissue transparency and light transmission. Similar to the brain, exchanges of molecules between retinal blood vessels and extravascular tissue are also very tightly regulated through the blood–retinal barrier (BRB).

Macular edema (ME) involves fluid buildup within or beneath the retinal layers in the macular area. This condition can arise from a range of retinal disorders and has the potential to damage central vision significantly. ME is caused by a disruption in the equilibrium between the influx and efflux of fluid, processes that are often impaired in retinal diseases and are influenced by multiple factors.^3^ ME can lead to a wide range of symptoms, including blurred or distorted vision and central vision loss, significantly affecting the individual’s quality of life.

In exudative macular edema (EME), retinal capillaries in the macula become leaky, leading to the accumulation of fluid in the tissue, forming cystoid spaces. Interestingly enough, fluid accumulation tends to occur mainly in the macular region, although the very center of the macula, the fovea, is avascular. The mechanisms of the selective accumulation of fluid in this very specific area of the retina are quite obscure and speculative, although the structure of the macular capillaries, together with the specialized morphology of foveal and parafoveal MCs, are likely to play a key role. To elucidate the role of these mechanisms, we propose here a mathematical model of fluid and solute transport in the retina.

Mechanistic mathematical modeling has proven to be a valuable tool for studying ocular physiology and pathology.^6^^–^^9^ Such models are designed to capture underlying physical mechanisms behind certain phenomena, making it possible to study their separate roles in physiological and pathological states. Several mechanistic models have been proposed for the retina.^10^ Roberts et al.^11^ developed a mathematical model to study oxygen transport in the retina and the role of neuroglobin in oxygen distribution across the retina. The authors also provided insights into the dynamics of oxygen diffusion, consumption, and delivery within the retinal tissue, particularly in the context of retinitis pigmentosa.^12^^,^^13^ To investigate in detail the role of retinal capillaries, Causin et al.^14^ presented a mathematical framework to simulate blood flow and oxygen transport in the retinal microvasculature and Chiaravalli et al.^15^ employed a multiscale/multiphysics model to investigate the configuration of retinal capillary plexuses and its potential influence on blood flow and oxygen delivery during various conditions. A mechanical model of exudative retinal detachment has also been proposed,^16^ which accounts for extravascular fluid accumulation in the subretinal space.

The present work complements previous studies, as we propose a mathematical model to describe fluid flow, protein transport, and tissue deformation in the retina in EME. A better understanding of EME pathophysiology may improve the management of such conditions and may stimulate the development of novel interventional strategies.

## Materials and Methods

### General Description of the Model

**Figure 1.**
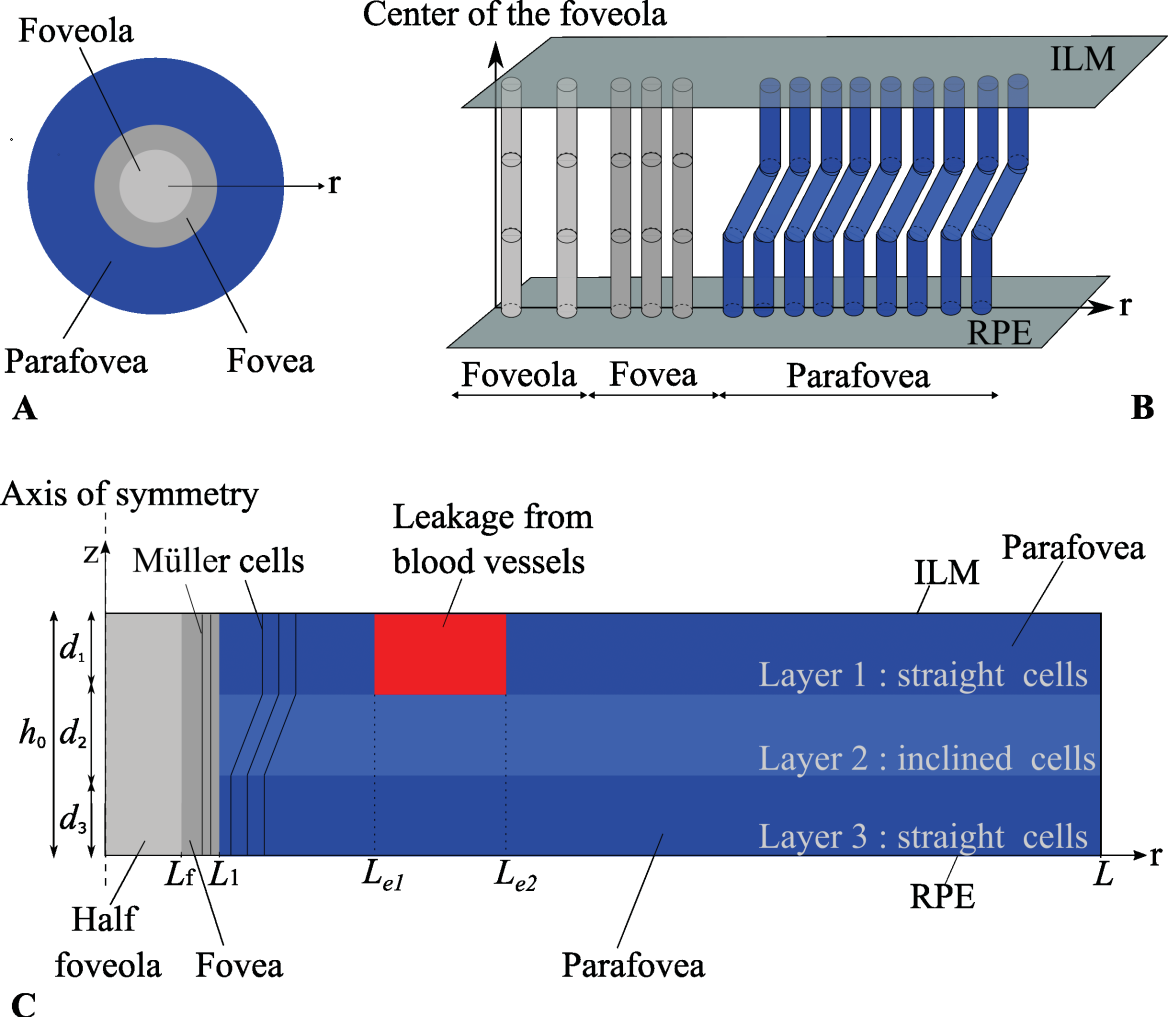
(**A**) Sketch of Müller cell density in the retina. (**B**) Spatial arrangement of MCs. (**C**) Sketch of the regions considered in the model. The red region denotes the exudation zone during exudative macular edema (EME).

The retina is described as an axisymmetric, non-homogeneous, porous medium. Since we consider a relatively small portion of the retina in the macular region, we neglect the curvature of the sclera. Adopting cylindrical coordinates (*r*, *z*), the *z*-axis is taken perpendicular to the retinal plane, and the *r*-axis is a radial coordinate that lays on the retinal plane, with the foveola located in correspondence with the axis of symmetry (*r* = 0), as shown in Figure 1. Thus, the *z*-axis passes through the center of the fovea and points toward inside of the eye globe. The thickness of the retina under the physiological conditions is assumed to be constant and equal to *h*_0_ = 320 µm.^3^^,^^17^ Thus, we do not consider the decrease of the retinal thickness in correspondence with the foveal pit for the sake of simplicity of the model. In the presence of EME, the retina deforms and takes a spatially variable thickness *h*(*r*). Though we also study the physiological case, the model described in the following is for the case of EME since it is most general.

We mentioned earlier that the mechanical properties of the retina strongly depend on the presence of MCs,^17^ the density of which is spatially variable. We account for this variability by splitting the domain into three regions in the radial direction, which are shown in Figure 1 with different colors: the foveola is depicted in light gray, the fovea in dark gray, and the parafovea in blue. We assign different values of tissue elasticity and hydraulic conductivity to these regions.

We also mentioned earlier that MCs are straight in the foveola and fovea and have a z-shaped conformation in the parafovea, where their extremities are orthogonal to the retinal plane, but their central parts are inclined, as schematically shown in Figure 1B. This inclination, which is, in reality, more exacerbated than shown in the figure, results in cells being closer to each other in the middle region of the retina (see Fig. 1B and the discussion in section “Homogenization-Based Approach to Estimate the Hydraulic Conductivity of the Retina” in the Appendix). We speculate that this affects tissue permeability, which we expect to be smaller where the cells are more tightly packed (i.e., where they are inclined). To account for this effect, we regard the retina in the parafoveal region as effectively further subdivided into three layers (in the *z*-direction) with different properties. These layers, denoted as 1, 2, and 3 in Figure 1C, are assumed to have the same thickness (*h*_0_/3) and different permeabilities to water. We estimate the relative permeability of each layer, making use of a homogenization approach, as discussed later on.

The retina is bounded on the inner side by the ILM and on the outer side by the RPE, as shown in Figure 1.

### Mathematical Formulation of the Model

We study fluid and albumin transport across the retinal tissue in physiological and pathological conditions. In physiological conditions, we assume that fluid flow is driven by a difference in oncotic pressure between the vitreous and the choroid and by the RPE active pumping. In pathological conditions, we take the BRB to be impaired so that additional fluid and protein leakage from retinal blood vessels occurs. When this happens, we assume that exudation primarily takes place in the internal region of the retina, where the retinal capillary bed is located.

We describe fluid flow as the flow in a porous medium and introduce a Darcy velocity **u**, defined as

(1) $$\begin{array}{c} u(x)=-K(x)\nabla p,\; \end{array}$$

where **x** represents the position vector, **K** is the hydraulic conductivity tensor (measured in m^2^/s/Pa), and *p* is fluid pressure. EME occurs over long time scales so that we can consider steady-state conditions. Mass conservation in steady-state conditions imposes

(2) $$\begin{array}{c} \nabla \cdot (K\nabla p)+q=0,\; \end{array}$$

where *q*(**x**) represents a source term due to fluid production (leakage from blood vessels into the extravascular tissue in the case of EME) and is taken to be a function of space. We assume that the source term *q*(**x**) is proportional to the difference between arterial capillary pressure *p*_*a*_ and the local tissue pressure *p* and that it is axisymmetric. Thus, we write it in terms of cylindrical coordinates as

(3) $$\begin{array}{c} q\left(r,z\right)=A_{f}\left(p_{a}-p\right)\frac{h_{0}}{h(r)}F_{r}\left(r\right)F_{z}\left(z\right).\; \end{array}$$

*A*_*f*_ is an amplitude coefficient (measured in s^−1^Pa^−1^), which can be interpreted as a measure of the hydraulic conductivity of blood vessel walls, while the dimensionless function $F_{r}\left(r\right)F_{z}\left(z\right)$ defines the region of impaired blood vessels and $\max\left(F_{r}\right)=\max\left(F_{z}\right)=1$. The functions $F_{r}$ and $F_{z}$ are chosen in such a way that exudation from blood vessels occurs in a ring region (*L*_*e*1_ ≤ *r* ≤ *L*_*e*2_ and (*d*_2_ + *d*_3_)*h*/*h*_0_ ≤ *z* ≤ *h*) in the most internal layer of the retina, which is schematically marked in red in Figure 1C, for the undeformed case. The function $F_{r}$ is taken as a double-step function, so that $F_{r}=0$ for *r* < *L*_*e*1_, $F_{r}=1$ for *L*_*e*1_ ≤ *r* ≤ *L*_*e*2_, and $F_{r}=0$ for *r* > *L*_*e*2_. The function $F_{z}$ is taken as a step, so that $F_{z}=0$ for *z* < (*d*_1_ + *d*_2_)*h*/*h*0 and $F_{z}=1$ for (*d*_1_ + *d*_2_)*h*/*h*0 ≤ *z* ≤ *h*. All these step functions are smoothed to avoid abrupt jumps. In Equation (3), we use the factor *h*_0_/*h*(*r*) so that the rate of fluid leakage from blood vessels does not depend on tissue deformation. The values of *L*_*e*1_ and *L*_*e*2_ are chosen so that the area of exudation is within the inner ring of Early Treatment for Diabetic Retinopathy Study (ETDRS) charts used to analyze diabetic retinopathy eyes. *A*_*f*_ is taken equal to zero in physiological conditions, whereas we prescribe a given value to model pathological fluid exudation.

For the transport of solutes in a porous medium, we use a steady-state advection-diffusion equation

(4) $$\begin{array}{c} \nabla \cdot \left(uc\right)-\nabla \cdot \left(D\nabla c\right)-q_{a}=0\; \end{array}$$

where *c* is the volume of solute per unit total volume, *D* is the diffusion coefficient, and *q*_*a*_(**x**) is the solute source term, representing the exudation of proteins from blood vessels.

We treat the protein solute source term similarly to what is done for the fluid term and write *q*_*a*_(**x**) as

(5) $$q_{a}\left(r,z\right)=A_{a}\left(p_{a}-p\right)\frac{h_{0}}{h(r)}F_{r}\left(r\right)F_{z}\left(z\right),$$

where *A*_*a*_ is an amplitude coefficient, and the spatial distribution is assumed to be the same as that of fluid leakage. As for the case of fluid leakage, we set *A*_*a*_ = 0 for the physiological case. In all simulations, we assume a fixed ratio between *A*_*f*_ and *A*_*a*_, such that *A*_*a*_ = *aA*_*f*_, and we take *a* = 0.5 mol/m^3^. This value of *a* is close to its theoretical upper limit, being albumin concentration in plasma equal to ≈0.53 mol/m^3^.^18^^,^^19^

Equations (1) and (4) are solved subject to the following boundary conditions. We assume that both the ILM and the RPE are permeable to the fluid, although with different permeabilities (larger for the ILM). Water flux through such membranes is regulated by Starling’s law, which accounts for both pressure-driven and osmotic fluxes. In addition, at the RPE, we consider the active cell pumping toward the choroid, *Q*_*RPE*_. These conditions are written as

(6) $$\begin{array}{ccc} & & \;-\left(K\nabla p\right)\cdot n=\Gamma_{RPE}\left[p_{c}-p-\sigma \left(\Pi_{c}-\Pi \right)\right]-Q_{RPE}\\ & & (z=0\;\text{at}\;\text{the}\;\text{RPE}), \end{array}$$

(7) $$\begin{array}{ccc} & & \;-\left(K\nabla p\right)\cdot n=\Gamma_{ILM}\left[p_{v}-p-\sigma \left(\Pi_{v}-\Pi \right)\right]\\ & & \;(z=h(r)\;\text{at}\;\text{the}\;\text{ILM}), \end{array}$$

where σ is the reflection coefficient, assumed to be equal to 1 for both membranes; Π is the osmotic pressure; the subscripts *c* and *v* refer to choroid and vitreous, respectively; and **n** is the outward normal vector. Finally, Γ is the hydraulic conductivity of the membrane, measured in m/s/Pa. The osmotic pressure is calculated using Van’t Hoff’s law, Π = *RTc*, where *R* is the universal gas constant, and *T* is the absolute temperature.

We assume that albumin can cross the ILM but not the RPE, owing to the presence of tight junctions between adjacent RPE cells; thus, proteins that leak out of blood vessels into the retina can only escape toward the vitreous chamber. The transport of proteins across the ILM is governed by Kedem–Katchalsky conditions.^20^^,^^21^ These conditions read

(8) $$\begin{array}{c} \;(uc-D\nabla c)\cdot n=0\;(z=0\;\text{at}\;\text{the}\;\text{RPE}), \end{array}$$

(9) $$\begin{array}{ccc} & & \left(uc-D\nabla c\right)\cdot n=\omega RT\left(c-c_{v}\right)+\frac{1}{2}\left(c+c_{v}\right)\left(1-\sigma \right)u\cdot n\\ & & \;(z=h(r)\;\text{at}\;\text{the}\;\text{ILM}), \end{array}$$

where ω is the membrane permittivity coefficient, measured in s · mol/kg/m.

The left boundary of the domain coincides with the symmetry axis (*r* = 0), and we impose regularity there. The right boundary (*r* = *L*) is arbitrarily set at a distance from the fovea that is large enough so that pressure and concentration are not affected by fluid and protein leakage in the macular region. We impose no-flux conditions there, for both the fluid and the solute.

Under physiological conditions, the retinal thickness is assumed to be constant and equal to *h*_0_. The physiological flow produces a physiological pressure *p*_0_(*r*, *z*) and concentration *c*_0_(*r*, *z*) distribution across the retina. In the simulations for the physiological case, the retinal tissue is considered rigid. We consider this as a “reference state” of the tissue.

Pathological fluid and albumin leakage from retinal blood vessels in EME generates an “excess” pressure and albumin concentration in the tissue. We assume that the retinal tissue deforms with respect to the reference state in response to this excess pressure. We also assume that the local tissue stiffness depends on the density of MCs and thus changes with the radial coordinate *r*. Details about how we treat tissue deformation and how we compute the elastic properties of the tissue are reported in the Appendix “Tissue Deformation.”

### A Simple Approach to Estimate the Hydraulic Conductivity of the Retina

Hydraulic conductivity (HC) is a measure of how easily a tissue lets a fluid flow through it. As explained above, we hypothesize that the conformation of MCs has a significant role in the HC of the tissue. We speculate that where MCs are inclined, they get closer to one another, thus decreasing the local tissue permeability to water. To quantify the importance of such an effect, we use homogenization theory^22^ to estimate the HC of the tissue.

We hypothesize that the resistance to water flow through the retina is mostly due to MCs, owing to their very high density in the macula. Thus, we ignore the presence of other retinal cellular populations, and we consider a creeping fluid flow in the space, assumed void, between MCs. We assume that MCs are straight, long, parallel cylinders regularly arranged in space. We relate cell spacing to their inclination: the more inclined cells are, the closer they get to each other.

Our homogenization approach allows us to compute the HC tensor of the tissue **K**, which appears in Equation (1), as a function of the cell inclination angle. Effectively, this means that we obtain an estimate of the HC in each spatial direction. This is done by numerically solving a problem for the HC tensor at the “micro-scale” (i.e., on a small, representative unit cell of the tissue), which is assumed to be periodically repeated throughout the entire tissue. More details on the method adopted are reported in the Appendix “Homogenization-Based Approach to Estimate the Hydraulic Conductivity of the Retina.”

We note that our approach implies that the HC predicted by the model is an overestimation of the real value since we neglect the presence of all retinal structures other than MCs. However, it allows us to understand how much HC changes with the inclination of MCs. In the Appendix, we show that the HC is ≈60 smaller for the inclined cells than for the straight cells. This is because, when the cells are inclined, they get so close to each other that they are almost in contact, thus obstructing fluid flow.

For simplicity, in our simulations, we assume that the HC in the three layers is isotropic (mathematically, this means **K** = *K***I**, with **I** the identity tensor). Extension to the anisotropic case presents no conceptual difficulty, and preliminary simulations showed that the results are very similar to those of the isotropic case. Thus, in this work, we do not discuss the effect of anisotropy and focus on the effect of different permeabilities in the three layers.

To correct the underestimated value of HC from the homogenization method, we did a thorough search of the available experimental measurements, as described in detail in the “Hydraulic Conductivity of the Retina, ILM, and RPE” section in the Appendix. The existing experimental data for these quantities are few and provide very sparse values, with sometimes two orders of magnitude difference between the measurements.^23^^,^^24^ A simple estimate of the pressure drop across the retina suggested that the value proposed by Antcliff et al.^24^ is the most plausible, as it results in a pressure drop of a reasonable magnitude. Therefore, we choose the value of *K* in such a way that the overall HC is 2.54 × 10^−10^ m/s/Pa^24^ (see Equation (15) in the Appendix for details).

**Figure 2.**
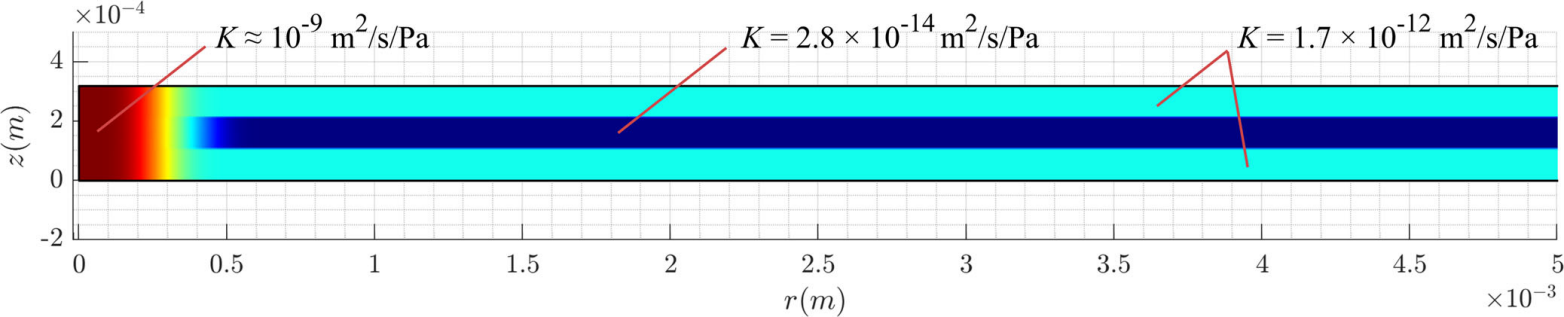
Values of the HC *K*, assigned in the various regions of the domain.

In Figure 2, we show the resulting hydraulic conductivity *K* in the retina. The value of *K* accounts for the different densities of the MC (see the Table) and the difference in HC in the different layers. Indeed, in the foveola, where the MC density is very small and the cells are straight, the HC is the largest. In the parafovea, where the cells are inclined, there is a difference of a factor of 60 between the middle layer and the outer ones. Throughout this article, we will use this map of *K* as our HC.

**Table.** List of Baseline Values of the Model Parameters

Parameter	Value	Units	Reference
Absolute temperature *T* at 37 deg	310	K	
Albumin concentration in the choroid *c*_*c*_	0.053	mol/m^3^	^26^
Albumin concentration in the vitreous *c*_*v*_	0.011	mol/m^3^	^25^
Diffusion coefficient *D* for albumin in a free solution	6.1 × 10^−11^	m^2^/s	^28^
Elastic modulus *E* of MCs	0.25	kPa	^29^
FAZ area in superficial retinal capillary plexus	0.289	mm^2^	^30^
Foveal hydraulic conductivity *C*_*f*_	1 × 10^−9^	m^2^/s/Pa	Assumed
Gas constant R	8.314	J/mol/K	
Membrane HC Γ_*RPE*_ of RPE	1.16 × 10^−11^	m/s/Pa	^31^
Membrane HC Γ_*ILM*_ of the ILM	1 × 10^−9^	m/s/Pa	^32^
Membrane HC of the retina	2.54 × 10^−10^	m/s/Pa	^24^
Length of the domain *L*	0.008	m	Assumed
MC density *n* in the fovea	10,000	cells/mm^2^	^33^
MC density *n* in the foveola	500	cells/mm^2^	^34^
MC density *n* in the parafoveal region	27,000	cells/mm^2^	^35^
MC diameter d_*c*_	5 × 10^−6^	m	^36^ ^,^ ^37^
Permittivity coefficient ω	1.97 × 10^−11^	s · mol/kg/m	Derived
Pressure in the arterial capillaries *p*_*a*_	25	mm Hg	^38^
Pressure in the choroid *p*_*c*_	0	mm Hg	Assumed
Pressure in the vitreous *p*_*v*_	0	mm Hg	Assumed
Radius of the foveal region *L*_1_	0.75	mm	^39^
Radius of the foveolar region *L*_*f*_	0.17	mm	^39^
RPE pumping *Q*_*RPE*_	1 × 10^−8^	m/s	^5^
Solute source term *A*_*a*_	0.5 (mol/m^3^) × *A*_*f*_	mol/s/m^3^/Pa	Assumed
Staverman reflection coefficient σ	1		Assumed
Stiffness of a single Müller cell κ_*c*_	≈4.9 × 10^−9^	N	Derived
Thickness of the vitreous cortex and ILM *d*_*m*_	120 × 10^−6^	m	^40^ ^,^ ^41^
Total thickness of the undeformed domain *h*_0_	320 × 10^−6^	m	^3^ ^,^ ^17^
Thickness of the bottom layer *d*_1_	*h*_0_/3	m	^3^ ^,^ ^17^
Thickness of the middle layer *d*_2_	*h*_0_/3	m	^3^ ^,^ ^17^
Thickness of the top layer *d*_3_	*h*_0_/3	m	^3^ ^,^ ^17^
Water source term *A*_*f*_	1.15 × 10^−4^	1/s/Pa	Assumed

### Numerical Methods

The problems on the microscale for the homogenization technique and the fluid Equation (1) and solute Equation (4) transport equations in the retinal tissue are implemented in weak form and solved numerically using the commercial software COMSOL Multiphysics version 5.6, which is based on the finite element method. Mesh invariance tests have systematically been performed using eight meshes, increasing the number of elements by ≈10% in each mesh, and sampling the computed pressure and concentration at five fixed locations to verify that the numerical solution did not depend on the numerical mesh. The mesh used for production runs showed a variation of less than 1% with respect to the most refined one.

## Results

In this section, we present the results of fluid and solute transport across the retinal tissue. In the following subsections, we discuss the results obtained for the “physiological case” and the “pathological case” corresponding to the formation of ME.

### Fluid and Solute Transport Across the Retina in the Physiological Case

In the physiological case, fluid flow is induced by the active pumping of the RPE and by an albumin concentration jump from the choroid to the vitreous. These effects generate a physiological fluid flow from the retina to the choroid. Fluid and solute exchanges between retinal blood vessels and extravascular tissue are not considered in this section.

**Figure 3.**
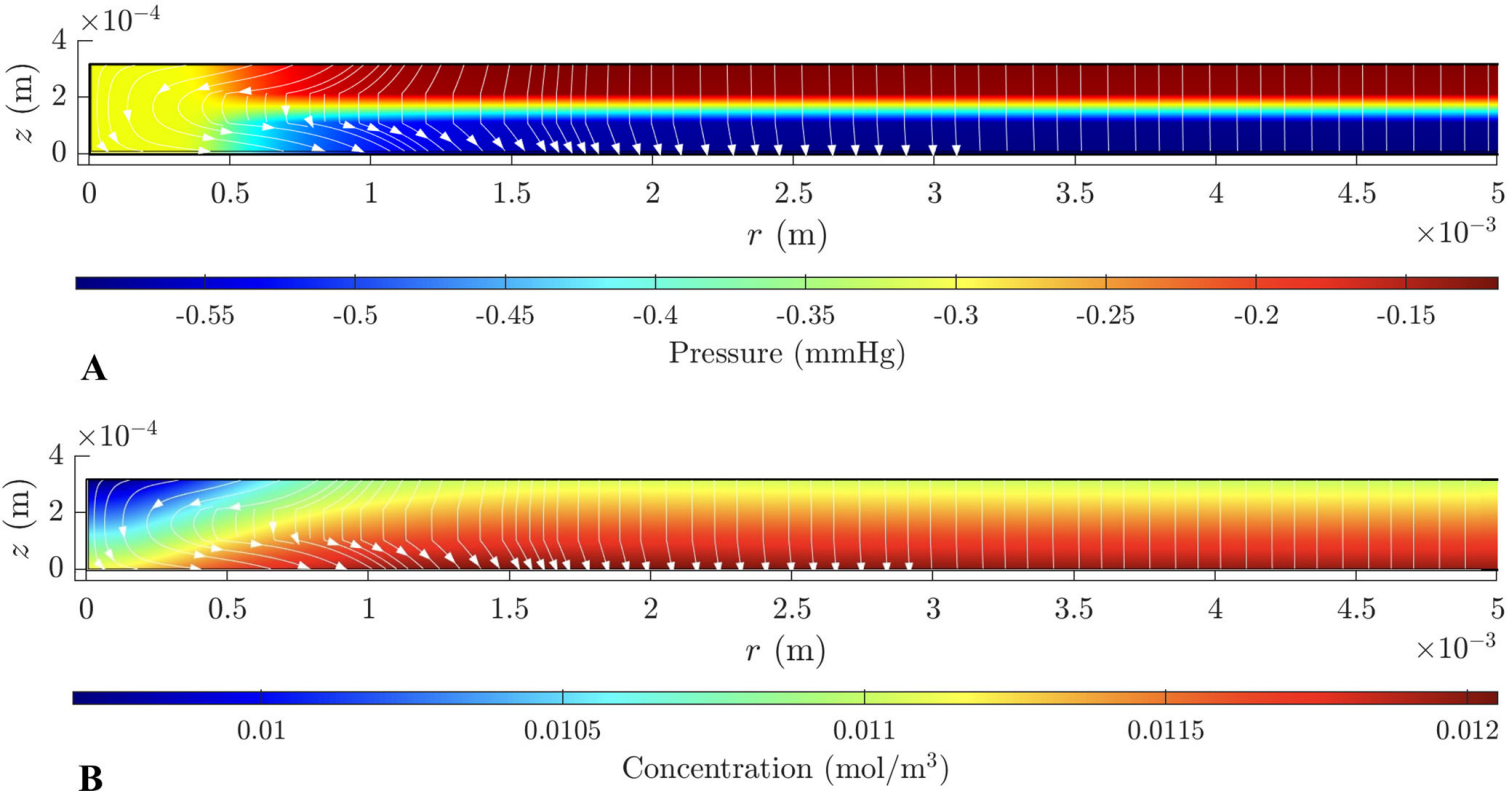
Pressure (**A**) and concentration (**B**) distributions and flow streamlines in physiological conditions.

Figure 3 shows the results of the physiological simulations. The figure’s plots refer to the pressure (A) and the concentration distribution (B). We assume that the interstitial pressure in the vitreous and the choroid is the same, and we set the values of 0 mm Hg with no loss of generality. This implies that the pressure predicted by the model should be seen as a departure from the interstitial pressure in the choroid and vitreous.

The albumin concentration in the vitreous is assumed to be equal to *c*_*v*_ = 0.011 mol/m^3^,^25^ while the concentration in the choroid is taken equal to *c*_*c*_ = 0.053 mol/m^3^.^26^ This difference in concentration induces fluid flow from the vitreous to the choroid, in addition to the flow generated by RPE pumping.

Figure 3A shows that the mechanical pressure difference across the retina is below 1 mm Hg. Most of the pressure drop occurs across the middle layer of the domain (layer 2), where the MCs are inclined, and the HC is 60 times smaller than in the other layers. The pressure remains nearly constant within the foveola (near *r* = 0), attributed to a significantly higher HC there due to the low density of MC and their straight alignment. This high HC also results in fluid velocities as high as 2.3 × 10^−7^ m/s, which is an order of magnitude larger than in the distal part of the domain (results not shown). The streamlines for the flow are shown with white lines and arrows. The flow is directed toward the choroid. The streamlines are curved near the boundary with the foveola, owing to the lower resistance there.

Albumin is transported by fluid flow and diffusion. The flow transports albumin toward the RPE, which is impermeable to it. Consequently, albumin accumulates in the lower part of the domain, leading to a buildup of its concentration; see Figure 3B. Far enough from the foveola (r⪆2 mm ), the solution for pressure and concentration becomes *r*-independent, and the velocity is one-directional across the retina.

The solutions shown in this section set the basis for studying the pathological case, which is seen as a departure from physiological conditions.

### Formation of Macular Edema

In the “pathological case,” we assume that fluid and albumin leak from blood vessels into the tissue. This induces additional fluid flow and albumin transport in the retina, and the excess pressure that is generated deforms the domain, creating the edema. Thus, we consider a coupled fluid–structure interaction problem in the full model. The exudation site is located at a distance between 1.33 and 1.83 mm from the center of the domain (*L*_*e*1_ = 1.33 mm and *L*_*e*2_ = 1.83 mm).

**Figure 4.**
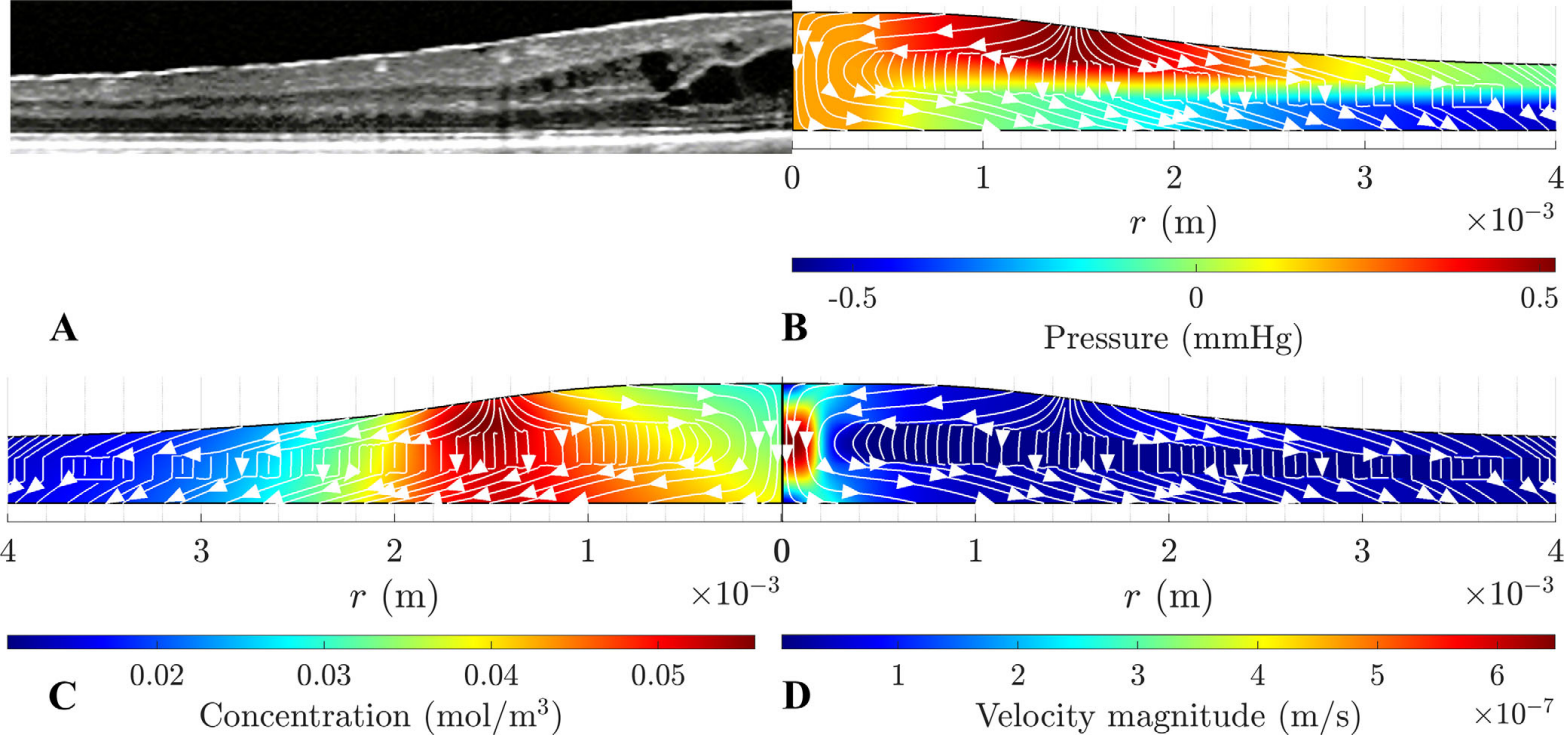
(**A**) OCT of the macular region in the presence of EME. (**B**) Pressure distribution. (**C**) Velocity field. (**D**) Concentration distribution. The *white curves* with *arrows* in (**A**–**C**) represent streamlines of the flow.

Figure 4 shows the results for the pathological case within a deformable domain. Panel A shows a medical optical coherence tomography (OCT) image of a retina affected by EME, and panels B to D show model predictions for the pressure, albumin concentration, and velocity. The OCT shows the deformation in the foveola and the surrounding parafoveal region; the maximum tissue deformation occurs in the foveola. The black holes in the image are cyst-like formations within the subfoveolar space (highlighted with red arrows in the figure), indicative of accumulated exudative fluid. Adjacent to this region, the retinal tissue maintains its physiological thickness. The predictions of the deformed retina in Figure 4B are qualitatively in line with the OCT image. The maximum deformation is obtained in correspondence with the foveal region, where fluid tends to accumulate, and the tissue is most deformable. In this region, the retinal thickness approximately doubles with respect to physiological conditions, which is a modeling choice in line with clinical observations, as discussed in the Appendix “Tissue Deformation.”^27^ Moreover, tissue deformation does not seem to affect the parafovea much.

Pressure distribution is shown in Figure 4B. Due to the exudation, the maximum pressure is higher than in the physiological case and peaks in the exudation region. Albumin is transported toward the RPE by advection and diffusion, where it tends to accumulate, hindered from further passage by the tight junctions in the RPE (panel C). In the region around the exudation site, albumin reaches the highest concentration of ≈0.05 mol/m^3^. Albumin exits into the vitreous through the ILM.

Panel D shows the fluid velocity and streamlines. Fluid is drawn from the vitreous at the exudation site due to the osmotic pressure differences caused by the buildup of albumin. The velocity magnitude is the highest in the foveola with the maximum of 6.5 × 10^−7^ m/s. Fluid exits the domain through the RPE. The fluid flow is significantly affected by the presence of a middle layer characterized by an HC approximately 60 times lower than the upper and lower layers, which creates an increased resistance. Indeed, the streamlines illustrate that fluid flows predominantly along the retina, except in the region close to the fovea. In regions sufficiently distant from the exudation site, pressure and velocity fields are congruent with the physiological case.

**Figure 5.**
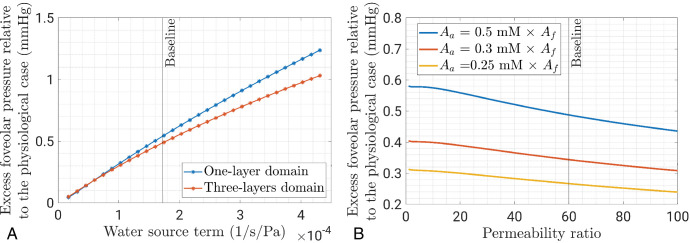
(**A**) Mean pressure in the foveola as a function of the fluid exudation rate. (**B**) Mean pressure in the foveola versus the HC ratio *K*^(1)^/*K*^(2)^ between the layer in which cells are straight and that in which they are inclined.

Figure 5A shows how the mean pressure in the foveola varies as a function of the magnitude of the fluid source term, *A*_*f*_ in Equation (3). The blue curve represents a model that depicts the retina as a single layer of a homogeneous porous medium, using an overall permeability value measured by Antcliff et al.^24^ In contrast, the red curve illustrates a scenario where the retina consists of three layers, each distinguished by its permeability, as for the prior simulations. In the figure, the magnitude of the fluid source term is varied from 10% to 250% of the baseline value, employed in prior simulations (see Figure 4 and referenced in the Table). The one-layer domain experiences a nearly linear increase in the average foveolar pressure with the water flow exudation rate. The three-layer domain exhibits a reduced slope compared to the one-layer domain. This reduced rate of pressure increase suggests that the presence of multiple layers mitigates the increase in tissue pressure in response to fluid exudation from blood vessels.

Figure 5B reports the average pressure in the foveola, in mm Hg, as a function of the HC ratio, defined as *K*^(1)^/*K*^(2)^, where the superscript (*n* = 1 and 2) refers to the layer *n*; see Figure 1. Thus, a permeability ratio set at 1 implies that the model comprises a single layer. This ratio has been changed while keeping constant the overall permeability of the retina, which is related to the permeability of the three layers according to Equation (15), as explained in the Appendix “Hydraulic Conductivity of the Retina, ILM and RPE.” The simulations for the three-layer model shown in Figure 4 have been run with a permeability ratio of 60. The three curves in the figure are for different albumin source terms. In all cases shown in Figure 5B, as the permeability ratio increases, there is a decrease in the foveolar pressure, indicating an inverse relationship between the two variables. At a value of the permeability of 100, the pressure is ≈0.76 of that obtained in a homogeneous tissue. MCs are inclined in the middle layer, with an inclination that can reach almost 90°.^17^ Thus, also based on the homogenization approach discussed earlier, we think that a reliable permeability ratio range lies between 50 and 100.

**Figure 6.**
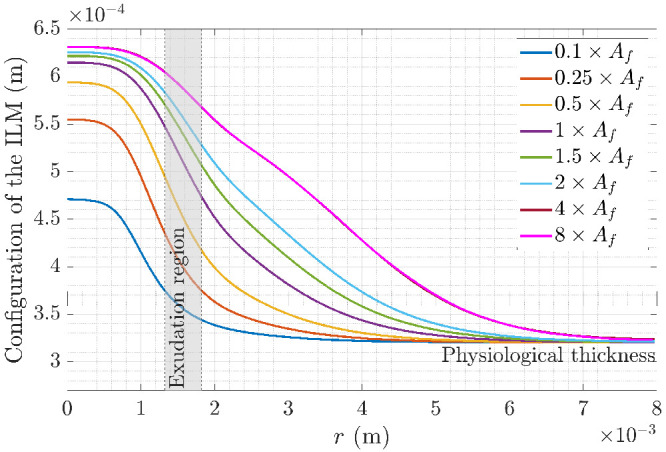
ILM profiles after deformation due to the exudation for different values of the fluid exudation rate amplitude *A*_*f*_.

Figure 6 shows the deformation of the ILM for different values of fluid source term (different curves). Each curve presents a maximum deformation in the foveola, which gradually decreases toward the periphery along the *r*-axis. Far enough from the foveola, the thickness returns to the physiological value. As the exudation rate increases, the deformation progressively expands laterally and extends within the parafovea.

## Discussion and Conclusions

This work proposes a mathematical model to describe fluid circulation in the retina, both under physiological conditions and in the presence of EME. This is the first attempt to develop a mathematical model of fluid flow and solute transport in the retina. Owing to the difficulties in directly measuring fluid and protein transport in the retina in vivo, a mathematical model has the potential to significantly improve our knowledge of the basic physical processes at play. Moreover, mathematical models can be helpful in understanding the role of specific effects, which might be difficult to isolate in experimental measurements. In fact, mathematical models have proven to be very informative in a variety of cases in the field of ophthalmology.^6^

We treat the retina as a porous medium with spatially variable permeability, which we assumed to be related to the variable density of MCs. We also hypothesize that tightly packed parafoveal MCs, which are oblique (z-shaped) in the middle retina, may reduce tissue permeability and obstruct fluid flow. To model this effect, we consider the retina as a three-layered structure, with each layer characterized by a different permeability, as illustrated in Figure 2.

Further, the retina is treated as a deformable tissue responding to the tissue pressure increase. MCs are known to have a critical role in maintaining retinal structural integrity by withstanding loads acting on the retinal parenchyma. For this reason, we assume that tissue stiffness varies spatially and proportionally to MC density, potentially causing increased tissue deformation at the foveal center, where MC density is much lower.

Another variable considered is protein transport in the retinal tissue and its consequences on fluid flow. To simulate BRB impairment, in our EME model, we assume that proteins and fluid leak from blood vessels in the innermost layer of the retina, where the retinal capillary plexuses are located. As leakage from capillaries must occur in the parafoveal region due to the avascular nature of the central fovea, specific mechanisms responsible for fluid accumulation in the center of the macula should exist. Our model aims to identify and explain such mechanisms.

Key parameters for the model are the values of the HC of the retinal tissue and the bounding membranes, ILM and RPE. Selecting values for these parameters has been very challenging due to the limited availability and inconsistencies among existing experimental data. A comprehensive description of our choices is detailed in the Appendix “Hydraulic Conductivity of the Retina, ILM, and RPE,” which may prove useful in future modeling endeavors.

Retinal HC is also estimated using homogenization theory, assuming that the density of MCs mostly determines the HC. As only MCs are considered, and other retinal cellular populations are not included in the model for the sake of simplicity, the calculated tissue HC is likely an overestimate. Our theoretical values are close to those measured by Fatt and Shantinath,^23^ but we consider these values far too high, as discussed in detail in the Appendix “Hydraulic Conductivity of the Retina, ILM, and RPE.” Therefore, we ultimately choose to use Antcliff et al.’s^24^ experimental measurement for the overall retina’s HC, but we retain a spatial variability of tissue conductivity that is based on our theoretical approach. As such, our HC theoretical analysis confirms that the inclination of MCs may have a major impact on tissue permeability.

Under physiological conditions, fluid transport in the retina is driven by active pumping of RPE and the osmotic pressure difference resulting from variations in albumin concentration between vitreous and choroid. The model incorporates these effects and predicts a water flux from the vitreous to the choroid, consistent with experimental observations.

In the pathological case of EME, when fluid and albumin exude in a specific region of the retina, we observe that the pressure peaks at the exudation site and progressively decreases away from it. However, tissue deformation also depends on the local tissue stiffness. The model predicts that deformation is maximal in the center of the fovea rather than at the point of maximum pressure, consistent with clinical observations. Thus, the model suggests that a key factor contributing to fluid accumulation in the central fovea is its greater compliance.

We also observe that the spatial variability of HC across the thickness of the retina, with the central region being less permeable to water, plays a protective role against fluid accumulation in the fovea. Specifically, we compare the results obtained by varying the ratio of HC in the central layer to the outer layer, finding that fluid pressure in the foveal region decreases as this ratio increases. In other words, if the tissue had homogeneous HC, the pressure in the fovea would be at its maximum, resulting in further thickening of the foveal region.

The model shows that the main driving force for fluid flow is osmosis, with mechanical pressure playing a minor role. For example, consider the pathological condition shown in Figure 4, where there is a maximum pressure increase in the tissue of ≈0.65 mm Hg compared to the physiological condition. This pressure increase goes down to ≈0.05 mm Hg if we switch off albumin leakage so that only fluid exudes from the retinal blood vessels but no proteins. This is because an increase in albumin concentration in the retinal tissue induces an osmotic flux from the vitreous chamber into the retina through the ILM.

The model also shows an increase of the RPE pumping hardly would have the potential to resolve fluid accumulation in the retina. Making again reference to the pathological case shown in Figure 4, a 3.5-fold increase of the RPE pumping decreases the excess pressure in the retina only by approximately 10%.

In conclusion, this work represents the first attempt to model fluid and protein circulation in the retina. Despite the various simplifications upon which the model is based and the uncertainty on some parameter values, our results can contribute to understanding the mechanisms that lead to the development of EME.

## Figures and Tables

**Figure A1.**
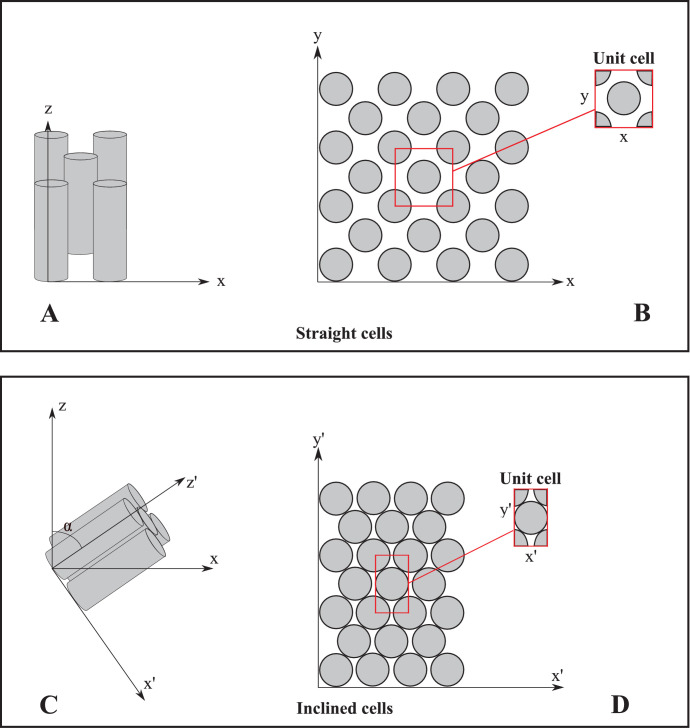
Sketch of the assumed arrangement of MCs for the calculation of the HC of the tissue and cross section of the micro-scale unit cells. In the top panel, MCs are perpendicular to the retinal plane. In the bottom panel, MCs are inclined at an angle α, and they get closer to each other.

**Figure A2.**
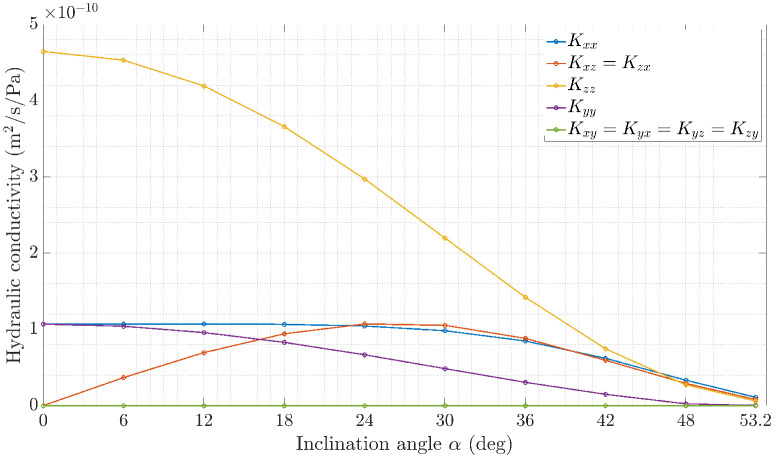
Dependency of the components of the hydraulic conductivity tensor *K*_*ij*_ on the angle of inclination of MCs. α = 0 refers to the case of cells orthogonal to the RPE plane.

**Figure A3.**
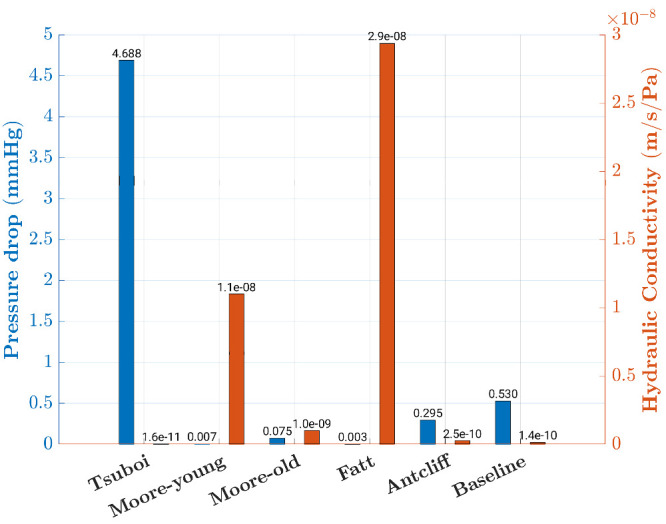
Bar chart displaying the relationship between hydraulic conductivity (m/s/Pa) and pressure jump (mm Hg) across the membrane for a water flux per unit surface of 1 × 10^−8^ m/s. Experimental measurements from Tsuboi^31^ (RPE–choroid complex), Moore et al.^43^ for young and old subjects (Bruch’s membrane and choroid), Fatt and Shantinath^23^ (whole retina), and Antcliff et al.^24^ The baseline bar refers to our choice and includes the contribution of the retina and the RPE–Bruch’s membrane complex.^24^^,^^31^

## Appendix

### Tissue Deformation

In physiological conditions, we treat the tissue as rigid, and we compute a spatial pressure distribution *p*_0_(*r*, *z*). We define a pressure averaged over the thickness of the retina as

(10) $${\bar{p}}_{0}\left(r\right)=\frac{1}{h_{0}}\int_{0}^{h_{0}}p_{0}\left(r,z\right)dz.$$

We denote with *p*(*r*, *z*) the value of the pressure in the presence of EME and with $\bar{p}\left(r\right)$ the corresponding depth-averaged value, defined as

(11) $$\bar{p}\left(r\right)=\frac{1}{h(r)}\int_{0}^{h(r)}p\left(r,z\right)dz.$$

We assume that the retinal thickness changes in response to the excess pressure $\bar{p}\left(r\right)-{\bar{p}}_{0}\left(r\right)$. In the numerical simulations, we use the following nonlinear law:

(12) $$\bar{p}\left(r\right)-{\bar{p}}_{0}=\frac{2\kappa \left(r\right)\varepsilon_{lim}}{\pi }\tan\left(\frac{\pi \varepsilon }{2\varepsilon_{lim}}\right),$$

where ϵ = (*h* − *h*_0_)/*h*_0_ is tissue strain, and ϵ_*lim*_ is the maximum admissible tissue strain, which we take equal to 1 since values above this (*h* > *h*_0_) are rarely observed clinically.^27^ The parameter κ(*r*) is the linear stiffness of the tissue (measured in Pa), which is taken to be proportional to the spatial density of MCs. We thus write

(13) $$\begin{array}{c} \kappa \left(r\right)=n\left(r\right)\cdot \kappa_{c} \end{array}$$

where *n* represents the varying density of MCs along the *r*-direction within the domain (measured in 1/m^2^), while κ_*c*_ denotes the stiffness of an individual cell.

We model MCs as cylinders, and we assume that when a single cell is acted on by a force of magnitude *F* in the longitudinal direction, it deforms according to *F* = κ_*c*_(*l* − *l*_0_)/*l*_0_, where *l* is the current length of the cell and *l*_0_ is the reference one. The stiffness of a single cell is defined as

(14) $$\begin{array}{c} \kappa_{c}=AE \end{array}$$

where *E* represents the Young’s modulus of the cell and *A* is the cross-sectional area of a single cell. The values of *n*, *A*, and *E* adopted are reported in the Table, with the corresponding references. With these numbers, assuming the cell density of the parafoveal region, we obtain the value of κ = 132 Pa. This is of the same order of magnitude as Young’s modulus of the retina as measured by Chen et al.,^42^ who found the values of 0.3 and 0.2 kPa for vertical and horizontal retinal strips, respectively. The value of ϵ_*lim*_ is chosen equal to 1 since values above this (*h* > 2*h*_0_) are rare, where *E* represents the Young’s modulus of the cell and *A* is the cross-sectional area of a single cell. The value of ϵ_*lim*_ is chosen equal to 1 since values above this (*h* > 2*h*_0_) are rarely observed clinically.^27^

### Homogenization-Based Approach to Estimate the Hydraulic Conductivity of the Retina

We model MCs as straight, long, parallel cylinders arranged regularly in an infinite lattice, as depicted in Figure A1.

We introduce a system of Cartesian coordinates (*x*, *y*, *z*), with the *z*-axis oriented perpendicularly to the retinal plane; thus, where the cells are straight, their axis is oriented along the *z*-axis. When cells are inclined, we suppose they are rotated by an angle α about the *y*-axis. We define another system of Cartesian coordinates (*x*′, *y*′, *z*′), such that *y*′ = *y* and *z*′ is in the direction of the inclined cells, that is, at an angle α with respect to *z* (Figs. A1C, A1D).

We consider a creeping fluid flow about the array of cylinders and use the homogenization approach proposed by Mei and Vernescu.^22^ At the macro-scale, this leads to Darcy’s law for fluid flow in a porous medium, given in Equation (1). At the micro-scale, the method requires considering a unit cell, which is periodically repeated. We choose a cell with arbitrary height in the *z*′ direction and the cross section shown in Figure A1. When the cells are straight (α = 0), we have *z*′ = *z*, and the unit cell has a squared cross section (top panel of Fig. A1). When the cells are inclined at an angle α ≠ 0, we suppose that the configuration can be obtained by rotating the cells while keeping fixed the intersection of their axis with the plane *z* = 0, as if they were hinged on that plane. This implies that the unit cell, which is a parallelepiped with sides oriented along the *x*′-, *y*′-, and *z*′-axes, has a rectangular base (see Fig. A1, bottom panel).

Following the approach of Mei and Vernescu,^22^ on the unit cell, we solve numerically a Stokes problem, which has a tensor field as an unknown, the spatial average of which represents the HC of the tissue **K**, which appears in Equation (1). Owing to the choice of the coordinate system adopted (*x*′, *y*′, *z*′), with *z*′ in the direction of the cells, the only nonzero terms of **K** are those on the main diagonal, $K_{x^{'}x^{'}}$, $K_{y^{'}y^{'}}$, and $K_{z^{'}z^{'}}$. The tensor components with respect to the straight coordinate system (*x*, *y*, *z*) are easily obtained by a rotation of amplitude α with respect to the *y*-axis.

We performed simulations varying the angle α from 0° to 53.2°, at which value the cells are so close as to almost be in contact with each other. The results are shown in Figure A2, where we plot each component of the tensor **K**, written with respect to the (*x*, *y*, *z*) coordinates, as a function of the angle α. When α = 0, we find that *K*_*xx*_ = *K*_*yy*_ and *K*_*zz*_ takes a larger value. As the angle α grows, the three components on the main diagonal progressively decrease, and the off-diagonal components *K*_*xy*_ = *K*_*yx*_ take nonzero values. The estimated HC in the *z*-direction decreases from 4.6 × 10^−10^ m^2^/s/Pa for α = 0 to 7.7 × 10^−12^ m^2^/s/Pa for α = 53.2°, which is approximately 60 times smaller. Even if we expect these values to overestimate the HC of the retinal tissue, our results clearly highlight the importance of MCs' inclination on the HC of the tissue.

### Hydraulic Conductivity of the Retina, ILM, and RPE

The HC of the retina and the two bounding membranes (ILM and RPE) are key parameters to understanding fluid motion and pressure distribution in the retina. Despite their significance, the existing experimental data for these quantities are few and provide very sparse values, this being particularly true for the HC of the retina. Choosing values for these parameters to be used in our model has thus proven to be exceedingly difficult. We therefore think that our choice deserves a thorough discussion. We also note that the terminology used in the literature is imprecise: with HC, people refer both to the conductivity of a tissue, **K** in the Darcy law (Equation 1) (measured in m^2^/s/Pa), and to the conductivity of a membrane, Γ that appears in conditions (Equations 6 and 7) (measured in m/s/Pa). There is a length difference between the two definitions: the membrane thickness. To avoid confusion, we refer to the latter as membrane HC.

To our knowledge, only two experimental studies of HC of the retina exist,^23^^,^^24^ which are separated by a considerable time and report highly discrepant results. Fatt and Shantinath^23^ measured the HC of retinas from enucleated rabbit eyes and found a value of 9.4 × 10^−12^ m^2^/s/Pa. Note that this value is similar to the one we obtained with the homogenization approach for the inclined cells (7.7 × 10^−12^ m^2^/s/Pa). Using a thickness of the rabbit retina of 120 µm, a value reported by the authors, Fatt and Shantinath^23^ found a value of 7.8 × 10^−8^ m/s/Pa for the membrane HC relative to the whole retina.

Thirty years later, Antcliff et al.^24^ measured the HC of the human retina in 41 pairs of human donor eyes, with a postmortem time varying from 24 to 56 hours. The membrane HC of the full-thickness retina was measured using a Ussing chamber, and the authors found the value of 2.54 × 10^−10^ m/s/Pa. This value is ≈300 smaller than the value found by Fatt and Shantinath.^23^ This huge difference is puzzling, even if measurements are taken on different species (humans and rabbits).

In addition to measuring the HC of the whole-thickness retina, Antcliff et al.^24^ performed a series of tests, progressively ablating the retina through its thickness, starting both from the vitreous and the RPE using an excimer laser. After each ablation step, the HC of the retina was measured using the Ussing chamber. Each ablation step increased the HC of the retina; however, the variation of HC was not homogeneous across the thickness, allowing the authors to identify high-resistance barriers to fluid movement in the retina. Oddly enough, the location of the barrier was found to be at a different location if ablation was performed, starting from the vitreous or the RPE. Despite the difficulty of interpreting this finding, the work by Antcliff et al.^24^ suggests that water barriers exist within the retinal tissue, which is in line with one of the assumptions underlying the present work.

Our domain is limited on the internal side by the ILM and on the external side by the RPE; moreover, Bruch’s membrane lies between the RPE and the choroid.

Studies on the HC of Bruch’s membrane and its changes with age have been conducted by Moore et al.^43^ The authors measured the HC of the isolated human Bruch’s membrane and choroid from eyes of various ages using a Ussing chamber. Results showed a significant, exponential decline in hydraulic conductivity with age, which is particularly pronounced in the macular region. In young donors, the macular HC was approximately 1.1 × 10^−8^ (m/s/Pa), decreasing to approximately 1 × 10^−9^ m/s/Pa in an 80-year-old donor.

Tsuboi^31^ investigated the membrane HC of the RPE–choroid complex in dogs and found a value of 1.6 × 10^−11^ m/s/Pa. We note that the author’s measurements combine the resistance to water flux of three separate tissues: RPE, Bruch’s membrane, and choroid. Among them, the contribution of the choroid is likely to be almost negligible.

Orr et al.^44^ studied the movement of intravitreally injected tritiated water from the vitreous to the choroid in rabbits. They observed that such movement was significantly accelerated by removing the intervening retina. Thus, they concluded that the main resistance to the motion of this tracer from the vitreous to the choroid is the retina. A similar conclusion was found by Kirchhof and Ryan,^45^ who also worked on rabbit eyes.

All measurements reported above are very poorly consistent with each other. To guide our choice of parameter values and get some feeling about the meaning of these numbers, it is useful to consider an elementary model of the flow through these membranes, driven by a pressure jump, so that *Q* = −ΓΔ*p*, where *Q* is the velocity across the membrane, Γ the membrane HC, and Δ*p* the pressure jump across the membrane. In Figure A3, we report with orange bars (right scale) the HC measured by the authors for the various membranes. With blue bars (left scale), we also report the corresponding pressure jump, computed assuming a *Q* = 1 × 10^−8^ m/s, which is a typical velocity associated with RPE active pumping.^5^ It appears that the predicted pressure jump varies from almost 5 mm Hg (which seems to be a large value) to the value of 3 × 10^−3^ mm Hg (which seems very small).

Orr et al.^44^ and Kirchhof and Ryan^45^ found that the retina offers a higher resistance to water flow than the RPE does. This finding can probably be regarded as quite robust since, in both cases, it is based on a comparative assessment of the HC of the tissue, with and without the contribution of the retina and performed on the same experimental setup.

As remarked above, the results obtained for the retina by Fatt and Shantinath^23^ and Antcliff et al.^24^ are very different. The pressure drop across the retina based on the value of HC of Fatt and Shantinath^23^ is extremely small and is not compatible with the findings of Orr et al.^44^ and Kirchhof and Ryan^45^ for any of the measurements of the RPE and/or Bruch’s membrane HC. We thus disregard Fatt et al.’s measurements and consider more reliable those by Ancliff et al., which are also more recent. Using the membrane HC of Antcliff et al.^24^, the pressure jump across the retina would be ≈0.3 mm Hg for the imposed water flux.

We note that even if we assume the membrane HC of the retina found experimentally by Antcliff et al.,^24^ which we denote as Kh, where *h* is the thickness of the retina (K measured in m^2^/s/Pa), we account for the fact that the tissue has inhomogeneous properties in the *z*-direction and consider the three layers of Figure 1, each characterized by a different HC in the *z*-direction. We can do this by combining the three layers in series to obtain

(15) $$K=\frac{\left(h_{1}+h_{2}+h_{3}\right)\left(K_{zz}^{\left(1\right)}K_{zz}^{\left(2\right)}K_{zz}^{\left(3\right)}\right)}{h_{3}K_{zz}^{\left(1\right)}K_{zz}^{\left(2\right)}+h_{2}K_{zz}^{\left(1\right)}K_{zz}^{\left(3\right)}+h_{1}K_{zz}^{\left(2\right)}K_{zz}^{\left(3\right)}},$$

where the superscript (*n* = 1, 2, 3) refers to the *n*th region across the thickness of the retina. In particular, (1) is the layer close to the ILM, (2) is the middle layer, and (3) is in contact with the RPE, as depicted in Figure 1. Strictly speaking, the above expression holds only for orthotopic tissues with axial symmetry with respect to the *z*-coordinate, but for simplicity, we adopt and make use of it in the following; even cells in layer (2) are inclined. Moreover, we set $K_{zz}^{\left(1\right)}=K_{zz}^{\left(3\right)}=60\;K_{zz}^{\left(2\right)}$ on the basis of our simulations (see section “Homogenization-Based Approach to Estimate the Hydraulic Conductivity of the Retina”).

If we accept the value K found by Antcliff et al.^24^ for the membrane HC of the retina, the results by Tsuboi^31^ provide too large a pressure drop and would contradict the observations by Orr et al.^44^ and Kirchhof and Ryan.^45^ The estimate of Moore et al.^43^ for elderly subjects would, on the other hand, be consistent with the observations of Orr et al.^44^ and Kirchhof and Ryan,^45^ even if they are only relative to Bruch’s membrane, and they disregard the contribution of the RPE.

In the simulations of this work, we have chosen a value of the HC of RPE and Bruch’s membrane together such that we obtain a pressure jump of ≈0.2 mm Hg, that is, smaller than the one across the retina based on Antcliff et al.’s value of the HC. This is approximately equivalent to increasing the HC of Tsuboi by a factor of 20.

The last bar in the histogram of Figure A3 shows our choice of parameters and includes the joint contribution of the retina and the RPE–Bruch’s membrane complex.

For the HC of the ILM, we could not find measurements. We have assumed that its permeability is much higher than that of the RPE and Bruch’s membrane because this membrane is extremely thin.

### Permittivity Coefficient

An important parameter is the permittivity coefficient, denoted as ω, that appears in the Kedem–Katchalsky boundary conditions Equation (9). This parameter quantifies the diffusive flux of albumin across a membrane (the ILM and the vitreous cortex, in our case). Owing to the lack of direct measurements in the literature, we estimate the ω using a one-dimensional Fick’s diffusion model and obtain

(16) $$\omega =\frac{D_{a}}{RTd_{m}},$$

where *d*_*m*_ represents the thickness of the membrane, that is, the thickness of the vitreous cortex and the ILM, and *D*_*a*_ is the diffusion coefficient for albumin. The thickness of the vitreous cortex is ≈110 × 10^−6^ m,^40^ while the thickness of the ILM was found to be ≈1 × 10^−6^ m.^41^ To represent the combined thickness, we select *d*_*m*_ = 120 × 10^−6^ m. Since albumin diffusion through the ILM and vitreous cortex is likely to be slower than in a free solution, we take *D*_*a*_ to be one-tenth of the corresponding value in a free solution (*D* = 6.1 × 10^−11^ m^2^/s). Thus, we get an approximate value of ω of 1.97 × 10^−11^ s · mol/kg/m.
